# Endophytic *Colletotrichum siamense* for Biocontrol and Resistance Induction in Guarana Seedlings

**DOI:** 10.1155/2021/1925226

**Published:** 2021-07-01

**Authors:** Luana L. Casas, José O. Pereira, Pedro Q. Costa-Neto, José F. Silva, Lucas N. Almeida, Roberto A. Bianco, João L. Azevedo

**Affiliations:** ^1^Postgraduate Programme in Biodiversity and Biotechnology—Bionorte, Amazonas State University (UEA), Higher School of Health Sciences, Manaus, Amazonas, Brazil; ^2^Federal University of Amazonas (UFAM), School of Agricultural Sciences, Manaus, Amazonas, Brazil; ^3^UNINORTE—Laureate International Universities, School of Health Sciences, Manaus, Amazonas, Brazil; ^4^University of São Paulo (USP), Luiz de Queiroz School of Agriculture, Piracicaba, São Paulo, Brazil

## Abstract

*Paullinia cupana* var. *sorbilis*, known in Brazil as guarana plant, is an important plant and a major traditional crop in the State of Amazonas. It is a native Brazilian species of great economic and social importance, particularly in the Amazon region. Anthracnose caused by *Colletotrichum* spp. is the main challenge for this crop. Therefore, the present study verified whether *C. siamense*, an endophytic fungus infected with a mycovirus, could protect the seedlings and reduce or eliminate the characteristic symptoms. Total proteins and enzymatic activities of pathogenesis-related proteins (PRPs), including peroxidase (POX), chitinase (CHI), and phenylalanine ammonia lyase (PAL), were quantified. Guarana seedlings of cultivar Maués were sprayed with a *C. siamense* conidia suspension (5.0 × 10^3^ conidia/mL). After ten days, the seedlings were sprayed with a suspension of the phytopathogen's conidia (1.0 × 10^6^ conidia/mL). One group of these seedlings received the fungicide indicated for this crop. The fungicide was applied twice with an interval of 15 days between applications. Negative control seedlings did not receive any treatment (except water and fertilization), and positive control seedlings were treated only with the phytopathogen. The experiment was conducted between December 2019 and February 2020 in a greenhouse. The treatments were applied at an average temperature of 25°C and 85% relative humidity. Leaflets were randomly collected from each treatment group at 0, 48, 72, and 96 hours after pathogen inoculation and analyzed for total protein and enzyme production (POX, PAL, and CHI). After 28 days, the percentage of leaf lesions on the seedlings was evaluated. *C. siamense* inoculation reduced lesions. There were differences in total proteins and PRPs at different timepoints after inoculation, except for CHI activity, among treatments. To the best of our knowledge, this is the first record of resistance induction in guarana plants.

## 1. Introduction

The guarana plant (*Paullinia cupana* Kunth. var. *sorbilis* (Mart.) Ducke), belonging to the Sapindaceae family, is a species native to rain forests. Its fruits are famous for their stimulating and other medicinal properties. This ethnopharmacological knowledge comes from indigenous communities and, most recently, from a local population [1]. Guarana powder, obtained from seeds via torrefaction and trituration, can be dissolved in water, ingested pure, or mixed with other herbal medicines [2]. The use of *P. cupana* has widely been explored in the soft drink industry [3], and it has also been used as raw material in the pharmaceutical and cosmetic industries in Brazil and elsewhere in the world [4].

*Colletotrichum* spp. cause anthracnose in guarana plants, characterized by leaf blight followed by defoliation. Under favorable conditions, acervuli develop inside the necrotic lesions, producing a conidial mass [5]. Mature or older leaves are not affected. Successive attacks of this fungus lead to the death of branches and, ultimately, of the plant [6]. Although various methods have been tested against pathogens, chemical control remains the gold standard to treat anthracnose [5].

Biocontrol is an alternative to chemical pesticide application because of its high efficiency, low cost, and environment friendliness, and it is increasingly being applied worldwide [7]. Physiological interactions between plants and microorganisms (fungi and bacteria) may bring benefits to agriculture, as these interactions promote plant growth and resistance [8].

Induced resistance is characterized as plant protection against phytopathogens using biotic and abiotic elicitors. Various microorganisms, such as fungi, bacteria, and viruses, can induce resistance in plants [9, 10]. Fungi have already been tested as biotic inducers in grape [11], cucumber [12], tomato [13], and barley [14].

*Colletotrichum siamense* Prihastuti, L. Cai & K. D. Hyde, an endophytic fungus of diverse hosts [15, 16], produces antimicrobial substances [17] and anticholinergics [18], and its potential as a bioherbicide has been explored [19]. In previous works [20], Casas et al. observed reduced anthracnose symptoms following the inoculation of *C. siamense* infected with a mycovirus at the time of planting in guarana seedlings of the BRS-Cereçaporanga clone.

To this end, the present study evaluated whether the endophytic fungus *C. siamense* induced resistance and reduced or eliminated typical symptoms in guarana seedlings of the BRS-Maués clone.

## 2. Materials and Methods

### 2.1. Microorganisms

Endophytic *C. siamense* carrying a mycovirus was previously isolated from healthy leaves of guarana plants at the Santa Helena farm in Maués, Amazonas (3°25′18.1″S, 57°40′40 8″W), and its potential for pathogen growth inhibition was analyzed *in vitro* [20, 21]. Pathogenic *C. fructicola* Prihastuti, L. Cai & K. D. Hyde was previously isolated from guarana leaves presenting with necrotic lesions and deposited in the Culture Collection of the Phytopathology Laboratory of the National Institute of Amazon Research (INPA). *C. fructicola* pathogenicity has been proven based on Koch's postulates [22].

### 2.2. Conidia Production for Analysis

*C. siamense* was cultured in test tubes for 7 days in synthetic nutrient agar (SNA) under an alternating light regime every 12 h at 30°C. In each tube, 10 mL of sterilized distilled water with 1% Tween 80 was added. Conidia were collected from the culture medium using a soft bristle brush and quantified using Neubauer's chamber. The suspension was adjusted to a spore density of 5.0 × 10^3^ conidia/mL. *C. fructicola* was cultured in potato dextrose agar (PDA) under the same conditions as above, and the suspension was adjusted to a spore density of 1.0 × 10^6^ conidia/mL using the standard applied to phytopathogenic *Colletotrichum* spp. [23, 24].

### 2.3. In Vitro Assay

#### 2.3.1. Seedling Preparation

Selected guarana seedlings of the BRS-Maués clone were kindly provided by Jayoro Agricultural Company (Presidente Figueiredo, Amazonas, Brazil). The substrate composition was humus plus sand (4 : 1), urea (0.56 kg/m^3^), KCl (0.3 kg/m^3^), and FTE-BR-12 (0.2 kg/m^3^) for mineral fertilization. Each black bag contained 3 kg of substrate, and the fertilizer was applied as top dressing every month following the relevant recommendations [6]. The seedlings were maintained in a greenhouse in the experimental area of the School of Agricultural Sciences (FCA) of the Federal University of Amazonas (UFAM). The experiment was conducted between December 2019 and February 2020. The experimental design was completely randomized with four treatments (negative control, positive control, biocontrol agent + pathogen, and fungicide + pathogen) and five replicates of 10 seedlings each. Seedlings that received only water and fertilization during the trial period were characterized as a negative control. Positive control seedlings were inoculated only with the pathogen.

#### 2.3.2. Biocontrol Agent Inoculation

Seedlings with three to four young leaves with fully expanded leaflets were used for this assay. Fungal suspension was applied to all leaflets (25°C, 86% relative humidity) using an electric pulverizer with a rotary compressor (40 lbf/pol^2^; Schulz, Brazil). A humid chamber was created using transparent plastic bags for 48 h. The bags were removed, and the seedlings were observed for 10 days before exposing to the phytopathogen. The negative control seedlings were sprayed with sterile distilled water alone.

#### 2.3.3. Preparation of Seedlings for Anthracnose Chemical Control

The seedlings used in this trial had between three and four complete leaves, with newly released, fully expanded leaflets. Seedlings used in chemical control assays were sprayed the fungicide flutriafol (12.5% m/v) prepared according to the manufacturer's instructions. The adhesive spreader Agral (2.5% v/v) was added to the mixture. An electric backpack sprayer (20 L) was used for application between 7 and 9 am (24°C, 90% relative humidity). The first spray was applied 2 days before exposure to the phytopathogen, and the second was applied 15 days after the first application (25°C, 86% relative humidity).

#### 2.3.4. Phytopathogen Inoculation

A *C. fructicola* suspension was prepared and adjusted to a concentration of 1.0 × 10^6^ conidia/mL. All seedlings, except the negative controls, were inoculated with the phytopathogen on the same day (25°C, 80% relative humidity) and incubated for 48 h in a humid chamber to facilitate conidial germination and colonization. The seedlings were evaluated daily for 28 days, according to the diagrammatic scale of anthracnose in guarana plants [25].

### 2.4. Enzymatic Assays

#### 2.4.1. Enzymatic Extract Preparation

Leaflets were randomly collected at 0, 48, 72, and 96 h post pathogen inoculation to measure total proteins and enzymatic activities of pathogenesis-related proteins (PRP). Five leaflets were collected from each treatment replicate. The leaflets (150 mg) were macerated in 1% (v/v) polyvinylpyrrolidone (PVP) and 1.2 mL of 0.1 M sodium acetate buffer (pH 5.1 mM EDTA) using a mortar. The extract was centrifuged (Centrifuge Excelsa® 4 280-R; Fanem, Brazil) at 17,970 × g for 25 min. The supernatant was transferred to microtubes and stored at −20°C [26]. All procedures were performed at 4°C.

#### 2.4.2. Total Proteins

Total protein content was determined using the Bradford method in a 96-well microplate [27]. Enzymatic extract (10 *μ*L) and Bradford's reagent (250 *μ*L) were added to each well. After 2 min (24°C), absorbance was measured at 595 nm using a spectrophotometer (SpectraMax Plus 384; Molecular Devices LLC, United States). Bovine serum albumin (BSA) was used as the standard, and the results were expressed in milligrams per milliliter [28].

#### 2.4.3. Peroxidase (POX) Activity

POX activity was determined at 30°C using a direct spectrophotometric method, based on the conversion of guaiacol to tetraguaiacol at 470 nm [29]. To 10 *μ*L of enzymatic extract, 290 *μ*L of a solution containing 250 *μ*L guaiacol and 306 *μ*L hydrogen peroxide in 100 mL of 0.01 M phosphate buffer (pH 6.0) was added. The mixture was added to the wells, and absorbance was measured after 5 min of reaction. Enzymatic activity was calculated using a molar extinction coefficient (*ɛ*) of 26,600 mol/cm, and the results were expressed in activity units per milliliter [30].

#### 2.4.4. Phenylalanine Ammonia Lyase (PAL)

To 30 *μ*L of the enzymatic extract, a solution containing 115 *μ*L of 0.1 M sodium borate buffer (pH 8.8) and 55 *μ*L of L-phenylalanine (20 mM) was added [31]. The mixture was allowed to react at 30°C for 30 min in a water bath. In control samples, the extract was substituted with 1 mL of sodium borate buffer. Next, 6 *μ*L of 6 N HCl was used to stop the reaction. The absorbance of trans-cinnamic acid derivatives was measured using a spectrophotometer at 290 nm. Enzymatic activity was calculated using *ɛ* of 104 mM/cm, and the results were expressed in activity units per milliliter [32].

#### 2.4.5. Chitinase (CHI)

CHI activity was analyzed using the dinitrosalicylic acid (DNS) method, based on the quantification N-acetylglucosamine (NAG) final reducer group with 1% (w/v) colloidal chitin as the substrate [33]. The method described by Miller [54] was used with the modifications [34]. To 0.035 mL of enzymatic extract, 0.035 mL of 1% colloidal chitin was added. The mixture was added to microplate wells and incubated at 50°C for 30 min. The reaction was stopped by adding DNS (0.1 mL) in a boiling water bath for 10 min. The samples were rapidly cooled to at 24°C by adding 0.08 mL of water and centrifuged (Centrifuge 5430 R; Eppendorf, Germany) at 2,204 × g for 10 min. Absorbance of the supernatant was measured at 540 nm. One unit of CHI activity was defined as the amount of enzyme that released 1 mol of NAG per minute under the described conditions. A standard curve was constructed using NAG (3.3 mg/mL), and the results were expressed in activity units per milliliter.

### 2.5. Statistical Analysis

The primary data were verified using a homogeneity test before analysis of variance (ANOVA). Results of different treatments were subjected to Tukey's test (*p* ≤ 0.05) using SISVAR 5.7 [35]. Results of lesions and enzyme activity were transformed to √(*x* + 1) and subjected to ANOVA after testing for homogeneity.

## 3. Results

### 3.1. Microorganisms

**Figure 1:**
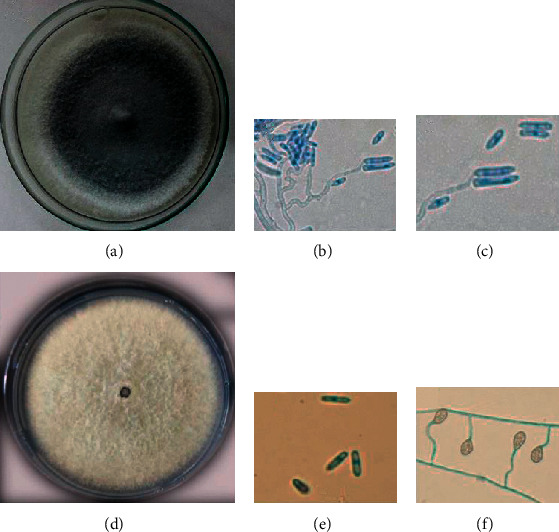
Morphology of the microorganisms used. (a) Morphology of the *Colletotrichum siamense* colony on potato dextrose agar (PDA). (b, c) Hyphae and conidia of *C. siamense*. (d) Morphology of the *C. fructicola* colony on PDA. (e, f) Conidia, hyphae, and appressoria of *C. fructicola*.

The endophyte *C. siamense* and phytopathogen *C. fructicola* (Figure 1) were cultured on PDA to obtain inoculants for guarana seedlings. The endophytic showed uniform growth and colony color typical of *Colletotrichum*. The phytopathogen had a faster mycelial growth, and although it did not show a mucilaginous mass of conidia, under the microscope it showed an intense production of them. Highlight for the presence of appressoria demonstrating the pathogenic characteristic of the fungus (Figure 1(f)).

### 3.2. In Vitro Assay

**Figure 2:**
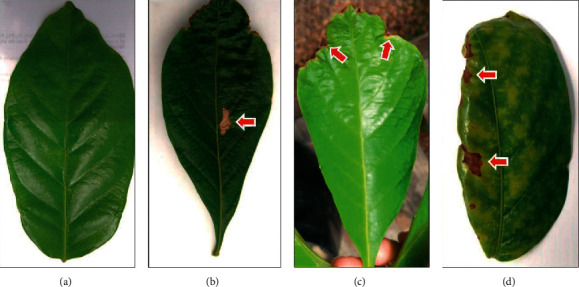
Guarana leaves under different treatments. (a) Negative control. (b) Positive control. (c) *C. siamense* + pathogen. (d) Fungicide + pathogen. The arrows indicate symptoms caused by the pathogen on the leaves 28 days after the start of the treatment.

The pathogen caused symptoms on leaves after 28 days after the start of the treatment (arrows, Figure 2). The first symptoms were observed from the fifth day after inoculation of the pathogen. Symptoms such as necrotic lesions on the leaves, reddish-brown in color, develop with greater predominance at the margin (Figure 2(d)), crusting of the leaf blade, and, in cases of severe infection, the total drying of the leaf. The percentage of symptoms of each treatment can be seen in Table 1.

### 3.3. Enzymatic Assay

Total proteins and PRPs were quantified in leaves collected at different timepoints. There were no significant differences in total proteins between the negative controls and infected seedlings until 96 h after phytopathogen inoculation (Table 2).

POX activity was higher in the infected seedlings than in the negative controls (Table 3). In the periods of 48 and 72 hours, the seedlings that had received the endophytic presented higher POX production than the seedlings of the negative control. After 72 hours, there was a drop in enzyme production in all treatments.

PAL activity was slightly increased after 48 and 72 h (Table 4).

CHI activity was slightly increased after 48 and 72 h (Table 5). CHI production was quite random when compared to other enzymes. Despite the statistical data showing a significant difference between treatments over 72 h, the difference in the number of enzymes was very small.

## 4. Discussion

In the present study, *C. siamense* was inoculated on guarana seedlings to evaluate its potential for inhibiting phytopathogens and altering total proteins and PRPs of the host. In anthracnose control assays, seedlings inoculated with *C. siamense* presented with a lower percentage of lesions than seedlings treated with a fungicide. However, the efficacy of biocontrol remained lower than that of disease control mediated by the plant's resistance mechanisms (Table 1). In previous studies, this same fungus was used to control anthracnose in guarana seedlings (“Cereçaporanga”), and a 5% lesion percentage was observed [20]. In the present study, the seedlings infected by the pathogen alone showed a lesion percentage of 1%; however, these values are higher in the field, reaching up to 50% in adult plants [36]. The present study was conducted at the seedling stage, for which there are no data indicating the percentage of loss due to anthracnose for comparison.

However, our observed results can be explained on the basis of the following points. In this study, the biocontrol agent suspension was used at a concentration of 5.0 × 10^3^ conidia/mL. In such assays, the inoculum must be more concentrated.

For instance, in studies with *Trichoderma asperellum* suspension [37], they used a suspension at a concentration of 5 × 10^8^ CFU to inoculate sorghum seeds, while in another study [38], a suspension at a concentration of 3 × 10^5^ conidia/mL was used. In addition, the number of biocontrol agent applications is determined based on the concentration of the suspension to be inoculated. Regarding the number of applications of the biocontrol agent, there are reports for other species of two applications with higher concentrations of the microorganism (10^8^ CFU) with an interval of 15 days [39].

Microorganisms have been recognized as potential resistance inductors and growth promoters. In *Camellia sinensis* seedlings treated with *T. asperellum* TC01, the severity of anthracnose caused by *C. gloeosporioides* was reduced by 58.2% [38]. In studies with *T. asperellum*, a reduction in the percentage of death caused by *Colletotrichum graminicola* (33%) was observed, in addition to an increase in the growth of sorghum [37].

Systemic resistance induced by microorganisms is an important characteristic of plant disease biocontrol [40]. POX, PAL, and CHI are associated with induced systemic resistance in the vegetal tissue [41]. To the best of our knowledge, the present study is the first to report on total proteins and PRPs in guarana plants. Average total protein content remained stable for 72 h in seedlings treated with *C. siamense*, but it slightly increased at 48 and 72 h following treatment with the pathogen alone (Table 2). In plants, protein synthesis occurs following the detection of microbial structures on vegetal tissue. These structures function as elicitors, activating a series of signaling molecules and inducing the expression of genes encoding PRPs [42]. The response time of protein synthesis varies according to the susceptibility or resistance of plant [43], phase of the plant life cycle [44], and type of elicitor (biotic or abiotic) [45, 46].

In the present study, the activity of POX and PAL was increased after 48 and 72 h in guarana seedlings infected with *C. fructicola* and inoculated with *C. siamense*. This increased enzyme activity may be attributed to the reduction of characteristic lesions, as POX is related to defense processes, including hypersensitivity response, lignification, suberization, and phytoalexin production [41]. In tobacco plants, a suspension of *Bacillus siamensis* was inoculated for the control of the phytopathogen *Alternaria alternata*. Maximum POX activity was observed 72 h after inoculation [47]. In chickpea seedlings inoculated with rhizobacteria as antagonists of the phytopathogen *Fusarium oxysporum* f. sp. *ciceris*, an increase in POX and PAL activity after inoculation was shown [48].

Inoculation of rhizobacteria in seeds promoted plant innate immunity and prevented symptoms, suggesting that the inoculation phase of the biocontrol agent may interfere with the efficiency of resistance activation against phytopathogens. PAL is the key enzyme in PRP synthesis. It catalyzes the nonoxidative deamination of phenylalanine to *trans*-cinnamic acid and ammonia, which is an initial step in the biosynthesis of phenolic compounds, including salicylic acid (SA) [49]. SA plays important physiological roles as a signaling molecule for inducing the expression of resistance genes against herbivores and pathogens [42]. There were no significant differences in CHI activity among treatments.

Chitinases are usually found in small amounts in vegetables, and their increase is seen after exposure to pathogens that have chitin in their structure, such as fungi, insects, and other invertebrates [50]. It was expected that, after inoculation of the pathogen, there would be an increase in the expression of this enzyme, which was not observed (Table 5). These results may be associated with the type of leaflet that was collected to obtain the enzyme extract. In the adopted methodology, the random collection of leaflets that were not always injured was standardized. Thus, it was inferred that during the observation of the plant's response to the presence of the pathogen was localized, the number of chitinases in healthy leaflets might not be as high. Besides, the activity of this enzyme *in vivo* is complex because it is associated with factors such as location and level of expression in plants [51]. For example, basic chitinases are located in vacuoles of plant cells and, possibly, will not come into immediate contact with fungi growing in the intercellular space [52]. Acid forms, on the other hand, are usually secreted into the apoplast or extracellular environment. Another hypothesis is that the plant's intrinsic resistance may have used other biochemical mechanisms to suppress the disease and not necessarily the production of enzymes such as chitinase. The plant's first line of defense is represented by structural and biochemical mechanisms that are present even before the pathogen is deposited. Cuticles, stomata, fibers, and trichomes are examples of structural mechanisms, while phenols, alkaloids, unsaturated lactones, cyanogenic and sulfur glycosides, phytotoxins, and proteins/peptides are preformed biochemical mechanisms [53]. This dynamic of factors interacting in synergy explains the nonhomogeneous distribution of leaf lesions that ranged from asymptomatic seedlings to others that were completely affected.

## 5. Conclusions

The present study demonstrated that *C. siamense* inoculation reduced the percentage of lesions caused by *C. fructicola* in guarana seedlings. In addition, the presence of this endophytic fungus promoted total protein and PRP synthesis at different times after phytopathogen inoculation. Additional studies are warranted to optimize the experimental conditions and validate the potential of *C. siamense* for biocontrol in guarana seedlings.

## Figures and Tables

**Table 1:** Average lesion percentage in guarana seedlings 28 days after the start of the treatment.

Treatment	Lesion (%)	CV (%)
Positive control	1 ± 0.023^a^	2.03
*Colletotrichum siamense* + pathogen	3 ± 0.044^a^	1.79
Fungicide + pathogen	4 ± 0.092^a^	2.38

Values in columns followed by the same letter are not significantly different (*p* > 0.05, Tukey's test). CV: coefficient of variation.

**Table 2:** Average total protein content of guarana leaves.

Treatments	Total proteins (mg·mL^−1^)				
	Elapsed time in hours after pathogen inoculation				
	0	48	72	96	CV (%)
CS + pathogen	0.002 ± 0.0007^b^	0.003 ± 0.0010^b^	0.003 ± 0.0010^a^	0.003 ± 0.0007^a^	0.32
*F* + pathogen	0.003 ± 0.0009^a^	0.002 ± 0.0006^a^	0.003 ± 0.0009^a^	0.003 ± 0.0009^a^	0.32
Positive control	0.003 ± 0.0009^a^	0.002 ± 0.0010^a^	0.002 ± 0.0012^b^	0.003 ± 0.0013^a^	0.46
Negative control	0.003 ± 0.0014^a^	0.003 ± 0.0011^b^	0.003 ± 0.0013^a^	0.002 ± 0.0011^b^	0.42

Values in columns followed by the same letter are not significantly different (*p* > 0.05, Tukey's test). CS: *Colletotrichum siamense*; *F*: fungicide; CV: coefficient of variation.

**Table 3:** Average peroxidase (POX) activity in guarana leaves.

Treatment	Peroxidase (U·mL^−1^)				
	Elapsed time in hours after pathogen inoculation				
	0	48	72	96	CV (%)
CS + pathogen	2.434 ± 0.39^a^	2.533 ± 0.34^a^	2.447 ± 0.38^a^	2.211 ± 0.46^a^	0.16
*F* + pathogen	2.030 ± 0.47^b^	2.325 ± 0.44^ab^	2.591 ± 0.29^a^	2.122 ± 0.42^ab^	0.18
Positive control	2.061 ± 0.46^b^	2.322 ± 0.35^ab^	2.577 ± 0.41^a^	2.182 ± 0.51^a^	0.19
Negative control	1.877 ± 0.49^b^	2.132 ± 0.52^b^	2.092 ± 0.45^b^	1.812 ± 0.52^b^	0.24

Values in columns followed by the same letter are not significantly different (*p* > 0.05, Tukey's test). CS: *Colletotrichum siamense*; *F*: fungicide; CV: coefficient of variation.

**Table 4:** Average phenylalanine ammonia lyase (PAL) activity in guarana leaves.

Treatment	PAL (U·mL^−1^)				
	Elapsed time in hours after pathogen inoculation				
	0	48	72	96	CV (%)
CS + pathogen	0.101 ± 0.02^a^	0.098 ± 0.01^ab^	0.099 ± 0.01^a^	0.087 ± 0.02^a^	0.20
*F* + pathogen	0.093 ± 0.02^a^	0.103 ± 0.02^a^	0.093 ± 0.02^ab^	0.084 ± 0.02^a^	0.23
Positive control	0.097 ± 0.02^a^	0.098 ± 0.02^ab^	0.085 ± 0.01^bc^	0.093 ± 0.02^a^	0.22
Negative control	0.085 ± 0.02^a^	0.087 ± 0.02^b^	0.072 ± 0.01^c^	0.079 ± 0.02^a^	0.24

Values in columns followed by the same letter are not significantly different (*p* > 0.05, Tukey's test). CS: *Colletotrichum siamense*; *F*: fungicide; CV: coefficient of variation.

**Table 5:** Average chitinase (CHI) activity in guarana leaves.

Treatment	CHI (U·mL^−1^)				
	Elapsed time in hours after pathogen inoculation				
	0	48	72	96	CV (%)
CS + pathogen	0.0002 ± 0.00005^a^	0.0002 ± 0.00003^b^	0.0002 ± 0.00004^a^	0.0002 ± 0.00006^a^	0.26
*F* + pathogen	0.0002 ± 0.00004^a^	0.0003 ± 0.00007^a^	0.0001 ± 0.00004^b^	0.0002 ± 0.00004^a^	0.27
Positive control	0.0002 ± 0.00005^a^	0.0003 ± 0.00006^a^	0.0002 ± 0.00006^a^	0.0002 ± 0.00006^a^	0.32
Negative control	0.0002 ± 0.00006^a^	0.0002 ± 0.00008^b^	0.0002 ± 0.0001^a^	0.0002 ± 0.00008^a^	0.37

Values in columns followed by the same letter are not significantly different (*p* > 0.05, Tukey's test). CS: *Colletotrichum siamense*; *F*: fungicide; CV: coefficient of variation.

## Data Availability

The data used to support the findings of this study are available from the corresponding author upon request.
